# From a Symptom-Based to a Person-Centered Approach in Treating Depressive Disorders in Adolescence: A Clinical Case Formulation Using the *Psychodynamic Diagnostic Manual* (PDM-2)’s Framework

**DOI:** 10.3390/ijerph181910127

**Published:** 2021-09-27

**Authors:** Annalisa Tanzilli, Guido Giovanardi, Eleonora Patriarca, Vittorio Lingiardi, Riccardo Williams

**Affiliations:** Department of Dynamic and Clinical Psychology, and Health Studies, Faculty of Medicine and Psychology, “Sapienza” University of Rome, Via degli Apuli 1, 00185 Rome, Italy; guido.giovanardi@uniroma1.it (G.G.); eleonora.patriarca94@libero.it (E.P.); vittorio.lingiardi@uniroma1.it (V.L.); riccardo.williams@uniroma1.it (R.W.)

**Keywords:** diagnosis, assessment, single case study, depressive disorders, PDC-A

## Abstract

Background: Depressive disorders in adolescence are among the most challenging clinical syndromes to diagnostically identify and treat in psychotherapy. The *Psychodynamic Diagnostic Manual, Second Edition* (PDM-2) proposes an integration between nomothetic knowledge and an idiographic understanding of adolescent patients suffering from depression to promote a person-centered approach. This single-case study was aimed at describing and discussing the clinical value of an accurate diagnostic assessment within the PDM-2 framework. Method: Albert, a 16-year-old adolescent with a DSM-5 diagnosis of major depressive disorder, was assessed using instruments from various perspectives: the Structured Clinical Interview for DSM-5; the Psychodynamic Chart-Adolescent of the PDM-2, and other clinician-report instruments; and the Shedler–Westen Assessment Procedure for Adolescents and Defense Mechanisms Rating Scale Q-sort, coded by external observers. Results: Albert’s assessment revealed impairments in various mental capacities, especially in regulating self-esteem. He presented a borderline personality organization at a high level and an emerging narcissistic personality syndrome. Conclusions: The case discussion showed the importance of providing clinically meaningful assessments to plan for effective treatments in youth populations. Especially, it is necessary to understand the adolescent’s unique characteristics in terms of mental and personality functioning and consider the developmental trajectories and adaptation processes that characterize this specific developmental period.

## 1. Introduction

Depressive disorders are among the most common psychiatric syndromes in adolescents worldwide, with an estimated prevalence rate of 4–5% found in mid-to-late adolescence [1,2,3]. Globally, depression is among the leading causes of illness and disability in young people (15–19 years), and a major risk factor for suicide. Notably, depressive symptoms seriously impact on social and psychological adjustments, exacerbating isolation and loneliness, increasing greater impairments in various domains of mental health, and raising the co-occurrence levels of self-harm, alcohol and substance misuse, anxiety disorders, eating disorders, and other clinical conditions [4].

Diagnosing depressive disorders in adolescence is more difficult than in adulthood. Evidence has shown that depressive pathologies in this specific development period represent a broad and more heterogeneous diagnostic grouping [5] and that, alarmingly, several cases of adolescent depression are not identified, accurately assessed, or dealt with in a timely and effectively manner. Thus, international health institutes and systems have recognized the urgent need to pay particular attention to early detection and prevention policies, as well as to the promotion of the best practices for the treatment of these disorders in adolescents, e.g., [6].

The *Diagnostic and Statistical Manual of Mental Disorders* (in the latest editions, DSM-IV-TR and DSM-5, [7,8] respectively; cf. also the *International Classification of Diseases,* ICD-11; [9]) defines major depressive disorder (MDD) as a clinical condition characterized by a pervasive sense of sadness and loss of interest and enjoyment in daily activities occurring most of the day, nearly every day, during a period lasting at least two weeks, along with difficulty concentrating, feelings of worthlessness or excessive or inappropriate guilt, recurrent thoughts of death or suicide, reduced energy or fatigue, and other symptoms that determine a clear impairment in an individual’s life. Similarly, the persistent depressive (or dysthymic) disorder (PDD) is described as a more chronic form of depression that can be diagnosed whether depressive mood and correlated symptoms lasts for at least 2 years. Despite the efforts of the DSM-5 Childhood and Adolescent Disorders Work Group to consider developmental extensions of the DSM-IV-TR’s depressive syndromes and symptoms, cf. [10], the current configurations of MDD or PDD do not show great changes; essentially, they remain more pertinent and closely applicable to adults, rather than to youth populations.

In recent years, increasing evidence has identified some specific characteristics of depressive disorders in adolescent populations, promoting a better understanding of the subjective experiences of these syndromes, such as the prominence of social isolation, loneliness, hopelessness and helplessness, irritability and hostility towards self and others, pessimism and despair, and withdrawal from the world [11,12]. Whilst these studies have provided insightful knowledge into the particular nature of youth depression, many relevant features of depressive affective, cognitive, and relational patterns in adolescents, except for irritability (see [8,9]), do not inform the diagnostic process grounded on symptom-behavior oriented approaches.

Another crucial issue in assessing youth depression is that several forms of these clinical conditions have been dismissed and viewed as normative crises inherent to the development stage [13] that reflects the adolescents’ difficulties in dealing with the complex and age-related changes at the physical, cognitive, social-emotional, and interpersonal levels. The healthcare professionals’ “dilemma” in distinguishing normative characteristics, which are associated with a period of life characterized by “storm and stress” [14] from persistent pathology, mostly arose from nosographic and diagnostic limitations. Actually, longitudinal studies examining depressive pathologies have supported meaningful differences between youth depressions and adolescent crises, highlighting that these psychopathologies co-occur with other forms of psychosocial impairment and, especially, predict not only mood disorders in adulthood, but also a wide range of other clinical syndromes [15].

Moreover, given the limited ability of various screening strategies in the early diagnosis of depression in youth populations, the need for investing in sophisticated diagnostic approaches and assessment procedures able to sensitively recognize warning depressive signs and symptoms and, in the meantime, evaluate the subjective experience of these clinical conditions in adolescents, has become urgent. Making accurate diagnoses and case formulations in adolescent populations is crucial to develop systematic therapeutic programs in order to reduce the burden of depression during this key developmental phase and prevent negative mental health outcomes across the lifespan [16].

The *Psychodynamic Diagnostic Manual, Second Edition* (PDM-2; [17]) is an international nosography aimed at addressing the limitations of the symptom-based diagnostic classification models by proposing an integration between nomothetic knowledge and idiographic understanding and promoting a person-centered approach. Aspiring to be a taxonomy of people rather than a taxonomy of disorders, the PDM-2 intends to both capture the complexity of clinical phenomena (functional understanding) and develop criteria that can be used in clinical and research settings (descriptive understanding). Its goal is to aid clinicians in understanding the mechanisms underlying patients’ psycho(patho)logical issues and planning patient-tailored treatments to enhance the efficacy and effectiveness of psychotherapies [18].

To favor complex and articulated diagnoses in young people, the PDM-2 adopts a developmental and comprehensive perspective to psychopathology [19]. Notably, it dedicates a specific section to adolescence and proposes a multidimensional and multiaxial approach that seeks to tap the individual’s overall functioning. This approach implies the assessment of mental functioning (MA Axis), personality organization and syndromes (PA Axis), and symptom patterns and their subjective experience (SA Axis). Unlike the multiaxial evaluation order in adults, where personality (P Axis) requires primary clinical focus (with respect to the M and S Axes), in adolescents, developmental issues related to mental capacities (MA Axis) have priority over the “emerging” personality patterns and psychopathological syndromes (PA Axis and SA Axis, respectively). Moreover, the PDM-2 highlights the importance of evaluating symptom patterns at the end of the diagnostic assessment, according to the assumption that, to capture the symptom’s function, it is critical to know something about the person hosting them [20]. From this perspective, the manual does not intend to replace the symptom-focused diagnostic systems, rather complement them [18,21]. Carefully assessing mental functioning and identifying emerging personality profiles in highly heterogeneous youth clinical populations are essential to foster personalized interventions in this developmental stage.

Noteworthy, research has found that personality pathologies are particularly frequent in adolescents with depressive disorders, e.g., [22,23,24]. These high levels of comorbidity suggest the need to investigate distinct personality subtypes in young patients suffering from depression. Although there are few studies in the field, some empirical investigations seem to confirm the presence of distinct styles/patterns of personality (especially borderline, narcissistic, avoidant, and dependent) both in adolescent and adult populations with depressive pathologies, e.g., [25,26,27]. Personality assessment in these syndromes is also very crucial, considering the significant impact of individual personological characteristics on treatment compliance and psychotherapy outcomes, cf. also [28,29].

Starting from these premises, in the current work we present the clinical case of Albert, a 16 year-old adolescent who presents with a DSM-5 diagnosis of MDD. Albert’s assessment described in the study is developed within the PDM-2 framework and is empirically supported by several tools that assist clinicians in their comprehensive diagnostic formulations and clinical reports. According to PDM-2, this research focuses on the multi-method and multi-informant evaluation of mental functioning, personality organization and emerging personality styles/syndromes, manifest symptoms and complaints, the subjective experience of symptoms, and other relevant cultural and contextual considerations. Notably, particular attention is paid to therapist responses towards the patient in psychotherapy (in this context, countertransference reactions; see also [30,31]). Indeed, the PDM-2 emphasizes the relevance of the clinicians’ subjective reactions in shedding light on their patients’ particular interpersonal modes and the psychological dynamics associated with specific personality patterns and psychopathological conditions [32,33,34,35,36,37].

The goal of this work is to discuss the clinical value of an accurate and empirically supported diagnostic assessment in capturing the complexity of Albert’s psycho(patho)logical functioning. We intend to stress the importance of considering a clinically sensitive perspective able to integrate the information provided by the DSM-5 approach with the knowledge derived from the psychodynamic assessment offered by the PDM-2. Overall, we aim to contribute to the current debate on clinical treatments for youth depression, highlighting that an accurate assessment of this syndrome is essential for effective treatment planning in adolescents.

## 2. Materials and Methods

### 2.1. Procedure

The case was randomly selected from a group of adolescent patients recruited in the context of a research project titled “Personality styles, mood disorders and therapeutic relationship in adolescent psychotherapy: A PDM-2 empirically oriented and clinically meaningful investigation” approved by the Ethical committee of the Department of Dynamic and Clinical Psychology and Health Studies, Faculty of Medicine and Psychology, Sapienza University of Rome, Italy (Protocol number: 0000232 del 01/03/2019). Following the ethical guidelines, all sensitive information in the case presentation were removed from the manuscript or kept confidential to guarantee the anonymity; thus, no explicit element allows the readers to trace and recognize the patient’s individual identity. Moreover, informed consent from the adolescent’s parents was regularly obtained.

Albert’s assessment was based on a wide variety of instruments from different perspectives: patient, therapist, and external observers. The initial evaluation called for a psychiatric intake interview of the patient and the administration of both the Structured Clinical Interview for DSM-5 (SCID-5; [8]) and the Personality Disorders Version (SCID-5-PD; [8]).

Albert’s clinician—a psychoanalytically-oriented psychotherapist with over 10 years’ experience and optimal expertise in using PDM-2 assessment in clinical contexts—was asked to complete a thorough evaluation of Albert with the Psychodynamic Chart-Adolescent (PDC-A; [38]) and other clinician-report instruments (see “Measures” section). Therapist first assessed the PDC-A and then, between one and three weeks later, other questionnaires in order to avoid potential evaluation biases.

To provide an empirical assessment of the patient from an external perspective, a group of four judges (all clinical psychologists and psychotherapists) were involved in the study. Albert’s clinician was interviewed using the semi-structured Clinical Diagnostic Interview (CDI; [39]), a set list of questions that offers systematic guidelines for obtaining comprehensive information from which to draw inferences about patients’ characteristic behaviors, affective states, emotion regulation processes, cognitive patterns, and implicit and explicit motives, fears, and goals. For research purposes, the CDI is widely used to increase clinical sensitivity while maintaining the advantages of standardized assessment protocols which facilitate high reliability and validity, e.g., [40]. This interview was audio-recorded, transcribed, and used in conjunction with other clinical material (three psychotherapy sessions selected from early in Albert’s psychodynamic treatment). External observers were asked to fill out the Shedler–Westen Assessment Procedure for Adolescents, Version II-A (SWAP-II-A; [24]) and the Defense Mechanisms Rating Scale Q-sort [DMRS-Q; [41]), two measures widely used in empirical literature and highly recommended in the adolescent section of the PDM-2. The judges (including two SWAP certified coders and two certified DMRS-Q raters) coded SWAP-II-A and DMRS-Q separately and blindly. The external raters’ group consisted of two women and two men; their average mean was 39.75 (range 37–43). They had about 10 years of post-training experience and performing of at least 10 h of direct patient care. Their theoretical-clinical approach was psychodynamically oriented. They compared their SWAP-II-A and DMRS-Q evaluations, and disagreements were solved through consensus discussions. Coders’ inter-rater reliability, determined using the intra-class correlation coefficient (ICC) on all SWAP items, was 0.78, whereas the raters’ ICC among all DMRS-Q scales was 0.81 on average.

### 2.2. Measures

Structured Clinical Interview for DSM-5 and the Personality Disorders Version (SCID-5 and SCID-5-PD): The SCID-5 and SCID-5-PD [8] were used for the assessment of DSM-5 diagnoses.

Psychodynamic Chart-Adolescent (PDC-A): The PDC-A [38] is a clinician report tool used to guide clinicians in the PDM-2-oriented diagnostic assessment of adolescents [17]. It starts with Section I: Mental Functioning (MA Axis), which provides an index of individual weaknesses and strengths by evaluating 12 capacities as: experiencing, expressing, and understanding different emotions; reflecting on one’s own and others’ mental states; forming and maintaining relationships; regulating self-esteem; using coping strategies and defenses, and so on. The clinician is asked to assess each of mental functions on a scale from 1 (*severe deficits or impairments*) to 5 (*healthy*), and then to provide an overall rating for the level of personality severity (as the sum of these 12 ratings). Section II: Level of Personality Organization (PA Axis) calls for ratings of identity, object relations, level of defenses, and reality testing similar to these ratings in the PDC-2, see [42]. Moreover, the clinician has to provide an overall rating of “emerging” personality pattern as either “normal” (*healthy*), mildly dysfunctional (*neurotic*), dysfunctional (*borderline*), or severely dysfunctional emerging personality patterns (*psychotic*). Section III: Emerging Adolescent Personality Styles/Syndromes (PA Axis) asks the clinician to evaluate the adolescent patient’s emerging personality patterns by checking as many relevant styles/syndromes as apply; then, the therapist notes the one or two most dominant patterns. Each pattern can be given a rating from 1 (*severe*) to 5 (*high functioning*). Section IV: Symptom Patterns (SA Axis) calls for ratings of psychopathological configurations coded by the DSM and ICD, but also asks the therapist to assess the adolescent’s subjective experience. Finally, Section V: Cultural, Contextual, and Other Relevant Considerations are similar to their counterpart in the PDC-2.

Therapist Response Questionnaire for Adolescents (TRQ-A): The TRQ-A [35] is an 86-item clinician-report instrument adapted from the adult version (TRQ; [32,43]), evaluating a wide range of thoughts, feelings, and behaviors expressed by therapists toward their adolescent patients. Clinicians assess each item on a 7-point Likert scale ranging from 1 (*not true*) to 7 (*very true*). In this study, we used an empirically supported TRQ-A version [37] consisting of six clinically sensitive patterns of therapist responses: *warm/attuned*, which describes an experience of close connection, trust, and collaboration with the patient (e.g., “I have warm, almost parental feelings toward him/her”; “I feel like I understand him/her”); *angry/criticized*, which indicates feelings of anger, hostility, and irritation, as well as a sense of being dismissed and devaluated by the patient (e.g., “I get enraged at him/her”; “I feel criticized by him/her”); *disorganized/frightened*, which describes feelings of being overwhelmed by the patient’s emotions and needs, and an intense sense of anxiety and dread toward the patient (e.g., “I feel I am ‘walking on eggshells’ around him/her, afraid that if I say the wrong thing s/he will explode, fall apart, or walk out”; “I feel anxious working with him/her”); *overinvolved/worried*, which indicates excessive engagement in the therapeutic relationship, including difficulties maintaining setting, and feelings of being critical of the patient’s parents (e.g., “I worry about him/her after sessions more than other patients”; “I talk about him/her with my spouse or significant other more than my other patients”); *disengaged/hopeless*, which describes a strong sense of frustration, inadequacy, and impotence, as well as feelings of boredom and withdrawal (e.g., “I feel hopeless working with him/her”; “I don’t feel fully engaged in sessions with him/her”); and *sexualized*, which indicates sexual tensions in the therapeutic relationship with the patient (e.g., “I feel sexual tension in the room”; “His/her sexual feelings toward me make me anxious or uncomfortable”). Reliability of TRQ-A scales show good internal consistency, with Cronbach’s α coefficient values ranging from 0.84 to 0.90.

Working Alliance Inventory–Therapist Version (WAI-T): The WAI-T [44,45] is a 36-item instrument that evaluates the pan-theoretical and multidimensional concept of the alliance that views it as an emotional bond that involves and supports collaboration between the patient and the clinician in the therapeutic work, along with their reciprocal agreement over the goals of treatment and the tasks necessary to achieve them [46]. The clinician rates each WAI item on a 7-point Likert scale, ranging from 1 (*never*) to 7 (*always*). The WAI provides a total score that defines the quality and strength of the alliance on the basis of its three dimensions or subscales: *goal*, which refers to agreement over the changes to be made in therapy (e.g., “We have established a good understanding between us of the kind of changes that would be good for my patient”; “We are working towards mutually agreed upon goals”); *task*, which refers to the methods and techniques used to achieve these outcomes (e.g., “My patient and I agree about the steps to be taken to improve his/her situation”; “I feel confident that the things we do in therapy will help my patient to accomplish the changes that he/she desires”); *bond*, which refers to the complex intertwinement of positive attachment between the patient and the clinician, involving mutual trust, acceptance, and confidence (e.g., “I feel I really understand my patient”; “My patient and I have built a mutual trust”). Research has provided robust support for the validity and reliability of the WAI, e.g., [45].

Shedler–Westen Assessment Procedure for Adolescents, Version II (SWAP-IIA): The SWAP-II-A [24]—the adolescent version of the SWAP-II [47]—is a psychometric instrument designed to provide a comprehensive assessment of adolescent personality and personality pathology. It includes 200 statements describing diverse domains of adolescent personality functioning, which are presented in jargon-free language common to clinicians of a wide range of theoretical orientations. A clinician or clinically experienced observer sorts (rank orders) the 200 items into eight categories, according to each item’s applicability to the patient, ranging from 0 (*not descriptive of the person*) to 7 (*most descriptive*), in order to comply with a fixed distribution. The SWAP-II-A furnishes: (a) scores for DSM-IV and DSM-5 personality disorder diagnoses or PD scales (used in the present study), and (b) scores for a taxonomy of adolescent personality styles/syndromes identified empirically through SWAP research or Q-factors (subdivided into three spectra of functioning–internalizing, externalizing, and borderline-dysregulated and an obsessional character style). It also includes a dimensional measure of psychological strengths and adaptive functioning. A personality disorder is assigned when the SWAP-200 assessment identifies one or more PD scale and/or Q-factor scores (in standardized *T* points) as ≥60 and a score on the high-functioning scale as ≤60. The SWAP demonstrated considerable reliability and validity in different settings and various clinical populations, e.g., [48,49,50]. Robust psychometric levels have been also obtained in multiobserver studies comparing diagnosis by treating clinicians with diagnoses by independent assessors based on research interviews, e.g., [51].

Defense Mechanisms Rating Scale Q-sort (DMRS-Q): The DMRS-Q [41] is a computer-based observer-rated measure developed for the assessment of defense mechanisms in clinical setting and based on the gold-standard hierarchy of defense mechanisms ([52]; see Table 1 for an overall description of the hierarchy of defenses).

The DMRS-Q consists of 150 statements describing 30 defense mechanisms in terms of personal mental states, relational dynamics, verbal and nonverbal expressions, self and others’ perceptions that emerge on occasions when the subject experiences internal or external stress or conflict. Raters must sort each statement utilizing a seven-point Likert scale, ranging from 1 (*least characteristic*) to 7 (*most characteristic*), which describes how each defense pattern contributed to the individual’s defensive functioning. After sorting all 150 statements into a seven-ranks forced distribution, the software provides a DMRS-Q report including: (a) a qualitative description of the patient defensive profile, so-called *defensive profile narratives* (DPN), and (b) quantitative scores for the ODF, seven hierarchically ordered defense levels and 30 defense mechanisms. The DMRS-Q has shown good levels of reliability and validity in adult and adolescent clinical populations [53,54,55].

## 3. Clinical Case

### 3.1. Case Illustration of Albert

Albert is a 16 year-old boy when he first comes to consultation, referred by a therapist who is to follow his parents for a couple therapy. It takes quite a long time before his mother contacts the therapist, just some days before the summer holidays. The mother is literally terrified by her son’s outbursts of rage and tells that, in one of the frequent family rows, the boy has just punched her husband face, breaking his lips. The woman reports feeling helpless and overwhelmed by the boy’s moody temper. She is entangled in the relationship with her son and seems unable to find a right distance and a useful key to understand him. She explicitly says she is not able to bear her son’s emotional demands and wants to get rid of this pressure. She finally entrusts the therapist with the charge of making Albert more “manageable” for the forthcoming period of holidays.

#### Personal History

Albert is the eldest son of a middle class family. His mother is a teacher in a secondary school and his father is a rather successful artist. Albert refers to having always breathed in a climate of exclusivity linked to his father’s aspiration. His father is presented as a kind man, socially isolated, and fundamentally committed to his artistic accomplishments. As a child, Albert was sent to a prestigious school far away from his residence and attended by the offspring of the local upper-middle class. He refers to having always felt as a “fish out of water” in that context, “a place that was not mine”, and started experiencing states of exclusion and inferiority toward his friends. Albert remembers that, back then, when his mother went to pick him up at school, he was ashamed of her and used to tell his friends that she was his au pair.

His parents have been facing a couple crisis for many years, being unable to share a real intimacy. They have been staying side by side without being really tied up to their relationship, without ever being mutually involved, and restraining their affective exchanges. Albert describes how both his parents seem to have had a special investment with him. A relationship nearly imbued with romantic shades, the one with his mother, in which he was called to occupy the place left vacant by his father, both from a psychological and physical point of view (when he started therapy he still used to sleep in the same bed with his mother). On the other hand, when confronted with her son’s emotional demands, the woman seems hardly sensitive to the boy’s requests for personal space and needs.

Albert describes the connection with his father as less affectively charged but still full of mutual high-standard expectations. The boy expresses the idea of being the depositary of his father’s cultural ambitions from which he draws a sense of intellectual superiority when “observing the facts of life”. He is really concerned about the possibility of not being recognized in this light of sensitivity and sophistication by the others (teachers and peers), who he often portraits as “simpletons”.

During the early stage of therapy, it emerged how the boy had always felt entangled between his parents’ conflict and, at the same time, sacrificed within the special role entrusted on him by both his mother and father. Notably, Albert acknowledges how this condition of apparent privilege precociously left him with the idea of having too little room left for the recognition and expression of his more intimate needs. On the other hand, what is harder for the boy to admit at the moment is how much he still clings to his special identity and how this perception affects his current interpersonal relationship both within and outside of the family. Thus, he frequently experiences bitter disappointments with his friends and romantic affairs when he discovers that his high standard of expectations is not met. He tends to over-react to these disillusions, oscillating between self-blame and harshly despising the others, or through violent outbursts of rage at home.

### 3.2. Clinical Case Assessment

#### 3.2.1. Symptom-Based Assessment from Patient Perspective

At the SCID-5, Albert met the criteria for the diagnosis of recurrent MDD. He complained of specific depressive symptoms such as: irritable mood; markedly diminished interest/pleasure in many activities; fatigue and loss of energy; feeling worthless or excessive/inappropriate guilt; sleep problems (insomnia); decreased concentration; and (sometimes) thoughts of death/suicide (cf. SA Axis of the PDC-A). Conversely, the SCID-5-PD did not indicate the presence of any personality disorder (cf. PA Axis of the PDC-A).

#### 3.2.2. Psychodynamic Assessment within the PDM-2 Framework from Clinician Perspective

Albert’s case has been evaluated using the PDC-A (Table 2) of the PDM-2. In the following paragraphs, the assessment is discussed in a detailed and comprehensive way.

SECTION I: Mental Functioning (MA Axis). Mental functioning is a set of processes measured by 12 capacities grouped in four main domains: (a) cognitive and affective processes; (b) identity and relationships; (c) defense and coping; (d) self-awareness and self-direction.

*Cognitive and affective processes* encompass the capacity for regulation, attention, and learning; the capacity for affective range, communication, and understanding; and the capacity for mentalization and reflective functioning. Albert shows a good mastery of the processes of regulation of the cognitive functions (attention, meta-cognition, memory, language and thought) and behavioral control (planning, inhibition of first response), as evidenced by his high school achievements and his articulated speech. However, impairments of these regulatory processes occur when facing interpersonal stressful events, affecting his concentration, capacity for reflection, and the modulation of emotional and behavioral expression that stems from negative affects.

*Identity and relationships* include the capacity for differentiation and integration (identity); the capacity for relationships and intimacy; and the capacity for self-esteem regulation and quality of internal experience. On the whole, Albert has a clear and refined view of the different emotional and thought nuances of the affective experiences between himself and others, showing authentic motivations for forming intimate and warm relationships. However, these capacities can be severely undermined when he feels a threat to his precarious self-esteem, shifting toward a black-and-white view of himself and of the others, alternatively oscillating between idealization or spiteful derogation, self-reliance, and clinging dependence, the fantasy of a total sharing of desires and mental states and a mutually persecutory contraposition. In these moments of disruptions, a self-referential preoccupation with his own needs emerges, and Albert does not appear anymore able to perceive the presence of others but for the constant seeking for approval and admiration.

*Defense and coping* encompass the capacity for impulse control and regulation; the capacity for defensive functioning; and the capacity for adaptation, resiliency, and strength. In the absence of interpersonal stressors, Albert presents a sufficiently mature organization of defenses and a rather flexible regulation of his emotions and impulses. Under these circumstances, he demonstrates a good compliance and a satisfactory adjustment to his developmental tasks and everyday challenges. When experiencing wounds to his self-esteem and rebuttals from the others, Albert can fall back upon a more rigid thinking with a poor capacity to employ his adaptive capacities to establish a personal control. He can gradually slip into impulsive and aggressive behaviors, or into a state of inner emptiness and emotional dysregulation.

*Self-awareness and direction* includes self-observing capacities (psychological mindedness); the capacity to construct and use internal standards and ideals; and the capacity for meaning and purpose. During the first therapeutic sessions, Albert revealed a good enough capacity to make connections between his personal history and current states of mind, in particular with reference to the childhood experiences of exclusion and humiliation at school and at home. Progressively, he was also capable of relating his arrogant, spiteful attitudes and persecutory feelings to the need to have his own needs accepted, as well as his sense of personal vulnerability: “I often feel lonely and hated by others but now I realized that I’ve left affective corpses on my wake”. It is only with difficulty that he recognizes his tendency to maintain an idealized relationship with his parental figures and a sense of personal superiority in other social contexts. As a fact, his intelligence and cultural background have allowed the boy to successfully pursue some objectives, developing a strong sense of personal autonomy. However, this aspect of self-reliance and self-sufficiency, while protecting him against delusions, has also declined into a sort of hardening in interpersonal relationships that he basically experiences in the light of personal achievements and competition.

Overall, the global index obtained in the first PDC-A’s section (score of 33) corresponds to moderate impairments in mental functioning (M04-High borderline level; range: 33–39). Albert shows several constrictions and inflexibility in most mental capacities, especially affecting self-esteem regulation, quality and stability of relationships, and the range of tolerated affects.

SECTION II: Level of Personality Organization (PA Axis). According to Kernberg’s [56] model, which strongly supports the PDM-2 framework, the level of personality organization is captured by assessing identity, object relations, level of defenses, and reality testing.

*Identity* is conceptualized as “the ability to view self in complex, stable, and accurate ways” [38]. Albert’s self-presentation is sufficiently rich and coherent in capturing both personal strengths and weaknesses. This vision has a temporal continuity: the boy is able to trace back intimate self-experiences (mainly referring to feelings of exclusion and shame) as early as from primary school. At the same time, Albert’s self-representation seems to be aiming at impressing and amazing the other so as to overshadow aspects of vulnerability. The boy interprets interpersonal disappointments, as well as the period of forced isolation due to the lock-down, as a personal failure. He may thus turn to torment himself with scathing self-criticisms, or devaluate what he describes as only apparently close relationships as actually unreliable and superficial. Often, he laments the fact of having been “betrayed” by those very people that he “had come to blindly trust.” Moreover, due to the strong reliance upon idealization, Albert tends to confuse the positive traits of his self-image with the other’s. When the sense of fragility takes over in his state of mind, he presents a disproportionally devalued self-image or, symmetrically, describes himself as a “victim, humiliated by cold and mean people around me”. Further, the boy is able to capture the bouts rage with his family, in which he “totally loses the temper”: “I transform myself into someone else I don’t recognize”. Overall, Albert’s functioning is characterized by a moderate level of identity diffusion, mainly justified by a “vertical splitting” between a hyper-valued and devalued sense of self worth and self/others’ relationships.

*Object relations* is defined as “the ability to maintain intimate, stable, and satisfying relationships” [38]. Albert shows a splitting between intra- and extra-familiar relationships that is rather typical of the adolescence functioning. In both contexts, recurrent aspects emerge that reflect the oscillation concerning self-images and the affective quality of intimate relationships. Indeed, within the family, he appears to be taken with a strong entanglement, in particular with his mother, or, alternatively, with a dismissing reaction that can be performed in a raging and resentful manner, especially when a frustration is encountered outside home. Friendships, as well as the only experience of falling in love he allowed to himself, reproduces the continuous and abrupt leaps from idealization to devaluation, where the other is blamed either for not being sufficiently committed to him or, on the other hand, for “not being up to” his special qualities or, worse, for having ridiculed or “disgraced” him in front of the others. He shows some difficulties in discussing the nature of his sexual interests that, at the moment, do not yet appear as adequately integrated in his developing identity. When interacting with adults, Albert is strongly oriented toward admirations seeking, exhibiting bitter disappointments, and may fall back upon discarding the meaning of the relationship (e.g., the learning rapport with teachers). This element becomes evident in his way to manage therapeutic relationships. Indeed, he tends to display his refined linguistic skills and literary and artistic tastes while downplaying the impact of his states of mind on the work and the relationship with the clinician: “you know what? I’ve finally decided to come back here, just because I’ve understood that I am able to analyze by myself some of the stuff we were discussing last time … so I now know I don’t need you for analyzing the dynamics … all I am asking you is to have some practical solutions to get out of all that shit.” On the whole, Albert seems to resort to an antagonistic and competitive attitude, whereas a sentiment of intimate sharing and a mature sense of dependence could emerge.

*Level of defenses* points out the ways in which the adolescent attempts to deal with and express wishes, affect, and other inner experiences, along with the ability to modulate anxiety resulting from internal and external conflicts, or threats to self without excessive distortion in self-perception and reality testing, and without making excessive use of acting out [38,52]. The defenses typically employed by Albert are addressed at the consolidation of his own fragile self-esteem and image, involving the denial of vulnerability, self-object idealization, the devaluation of the object aiming at denying both the lack of recognition from the object as well as the dependence from the other for his own self-esteem. Being brought up in a well-educated family, the boy is able to boost his image through the defense of rationalization and intellectualization, through which he is used to presenting himself while seeking for recognition in a social context, thus adhering to the father’s idealized expectations. Defenses of acting out are also present, though in limited moments of his everyday life and being frequently triggered by the family tensions that have reached a peak in the last year. On the other hand, under conditions of less emotional pressure, Albert seems able to achieve a more integrated and realistic representation of self–other relationships.

Finally, *reality testing* is “the ability to appreciate conventional notions of what is realistic” [38]. Albert preserves the capacity to distinguish the perceptual differences between the self and the other. He answers to the questions in a consistent, punctual, and articulated manner, and he is able to make pertinent reflections upon the themes that emerge in the dialogue. Retrospectively, he appears to acknowledge the distortion of the meaningful interactions emerging in periods of emotional turmoil. However, he reports losing this capacity under stress or when he receives critiques or does not feel adequately recognized by the others. As far as his relationships with teachers in school are concerned, he may feel prosecuted when he receives a bad mark: “I am sure they make it on purpose, just because they hate me, they dislike me and won’t understand what I can really express”. Moreover, he feels humiliated by his peers: “they are untrustworthy and lousy, they’ll never understand who I really am”. Although Albert provides a clear enough demonstration of a *repertoire* of obsessive and neurotic defenses, he may also rely on immature defenses, showing momentary declines in his capacity for the self-observation of emotionally polarized representations (that stem from splitting between idealization and devaluation).

Overall, a borderline level of personality organization (high level; [57]) is to be considered. This level of personality organization accounts for a moderate level of identity diffusion revolving around an unstable regulation of his self-esteem, transient moments of emotional dysregulation and aggressive acting out, and fleeting mood conditions, as evidenced through the depressive symptoms (cf. also Axis SA).

SECTION III: Emerging Personality Styles/Syndromes (PA Axis). The narcissistic pattern is the most dominant of all. Albert was brought up in a context in which he had a pivotal function in supporting his parents’ mutual disappointments and narcissistic vulnerability. He seems to have developed a global attitude of over-compliance toward external demands to the detriment of the capacity to feel recognized and to establish an intimate communication with meaningful others. He then tends to experience any aspect of dependence and realistic vulnerability as personal failures or signs of unbearable inner weakness. Albert has learnt to deal with this inner threat with a grandiose and unquestionable self-representation, an extreme idea of self-reliance and admiration-seeking behaviors. When the boy experiences the inevitability of disillusion and a relative wound to his self-esteem, he can resort upon physical, mental, and verbal forms of self- or hetero-directed aggression from which he regains a sense of self-power. By the same token, he has a peculiar way of presenting his suicidal ideation as a fantasy of escape from an unbearable situation created by feelings of personal failure. This sibylline communication seems aimed at producing the effect of generating alarm and subtly establishing a control over others when feeling too dependent. When seeking for a close relationship or facing moments of loneliness, the boy nearly exclusive relies upon the recognition of his unique intellectual brightness, cultural superiority, physical beauty. However, when his need for admiration and validation is frustrated, severe self-criticisms gain the upper hand, favoring intense depressive falls.

*Countertransference reactions and therapeutic alliance*. In line with the PDM-2 framework, countertransference reactions and the quality of therapeutic alliance were explored to increase clinicians’ knowledge of the patients’ modes of relatedness. The use of TRQ-A and WAI-T (see “Measures” section) was helpful to identify distinct affective, cognitive, and behavioral experiences of the therapist working with Albert, as well as to evaluate the characteristics of their interpersonal connection. In more detail, the TRQ-A results pointed out the prevalence of disengaged/hopeless (3.00), angry/criticized (2.50), overinvolved/worried (2.57), and warm/attuned (2.73) countertransference patterns, whereas the WAI-T global score showed a moderate level of therapeutic alliance (3.50). Overall, Albert’s diagnosis of emerging narcissistic personality syndrome seems to be empirically supported. As reported in the PDM-2 [58], in general, therapists may respond positively to (and be seduced by) narcissistic patients, but they may experience negative countertransference reactions (rage or boredom) when they feel unappreciated or when interacting with aspects of a patient’s false self [59]. Albert’s clinician experiences a complex mixture of feelings to Albert that strongly reflects specific personality features of the patient. At the same time, he feels a good engagement in therapy.

SECTION IV: Adolescent Symptom Patterns: The Subjective Experience (SA Axis). This section focuses on adolescents’ subjective experience of symptom patterns as described by the international classifications of mental disorders. Albert describes the presence of symptoms of anxiety and depression that are often accompanied by temper tantrums, in which he may become physically and verbally aggressive toward his parents and brother. The symptoms seem to have worsened after the beginning of the pandemic and have also been followed by a progressive withdrawal from social contact with his peers.

Confirming the SCID-5 results, Albert presents with irritability; anxiety with the sensation of being on the edge; feelings of loneliness and hopeless about his future; distractibility; sleep disturbances (he has a strong difficulty in falling asleep and needs smoking hashish to sooth himself); and a covert suicidal ideation is also reported as “bad thoughts that I make at night when going to bed.” The diffuse state of irritability and aggressive impulses are described as appearing at the beginning of adolescence and experienced as an unbearable condition that he experiences as meaningless and makes him feel out of control. Notably, the suicidal ideation is mild in intensity and not recurrent; it intensified by conditions of social isolation due to the lock-down or to more conspicuous external frustrations (with peers or in the context of school) that are experienced as irreparable personal failures in peculiar personological functioning [60].

Moreover, according to Blatt’s [28] model, which distinguished between two kinds of depressive affects and configurations (see SA Axis; [61]), Albert seems to show the features of the “introjective” (or self-critical) form, which concerns the achievement of a positive and cohesive sense of self. Albert’s subjective experience of depressive pathology, indeed, is characterized by feelings of failure, emptiness, and self-criticism; moreover, the suicide ideation is one of the symptoms of this depressive configuration cf. also [62,63].

#### 3.2.3. Empirical Assessment from External Perspective

In order to integrate Albert’s assessment from the clinician, external raters completed the SWAP-II-A [24] to assess personality traits and pathology, as well as the DMRS-Q [41] to evaluate defensive functioning based on the whole hierarchy of defense mechanisms ([52]; cf. Table 1).

**Figure 1:**
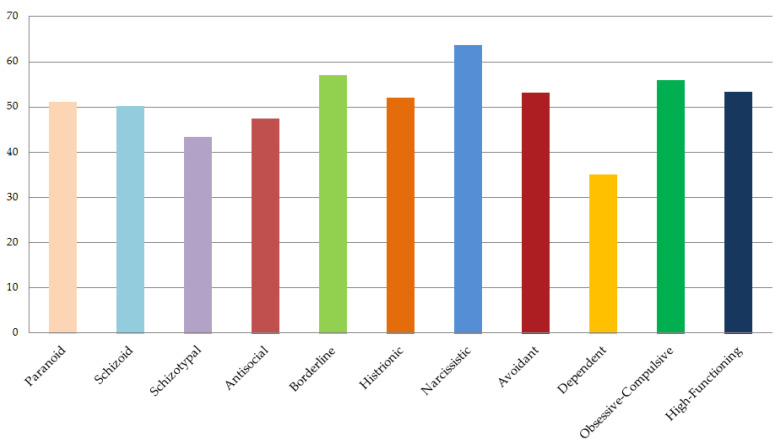
The personality profile (PD scales) derived from the SWAP-II-A shows that Albert presents an emerging narcissistic personality syndrome and an average high-functioning score.

Figure 1 depicts Albert’s personality profile in relation to PD scales of the SWAP-II-A. The presence of a narcissistic personality syndrome (T = 63.74) was confirmed. Moreover, it is important to highlight borderline (T = 57.12) and obsessive personality traits (T = 56.85), as well as an average high-functioning score (T = 53.40) (see Supplementary Materials in Table S1).

**Figure 2:**
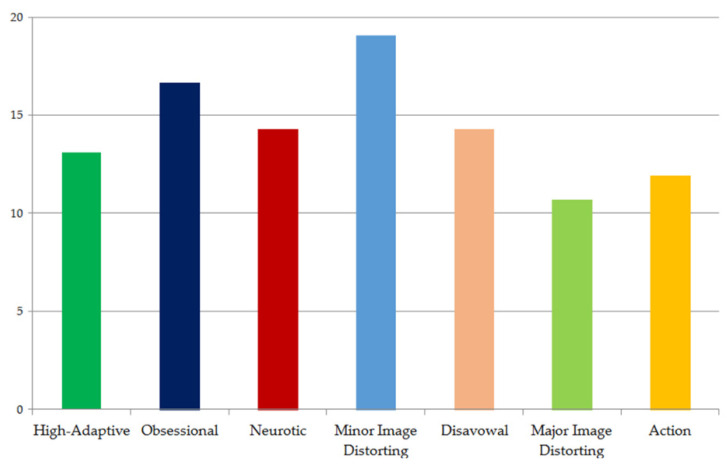
The defensive profile empirically derived from the DMRS-Q shows that Albert mostly uses the minor image-distorting defenses.

Figure 2 shows the profile of Albert’s defensive functioning empirically derived from DMRS-Q. In line with the clinician’s perspective, the results highlighted the prevalence of minor image-distorting defenses, along with the use of both obsessional and immature defenses (see Supplementary Materials in Table S2).

Overall, Albert deals with emotional conflicts, or internal or external stressors, by attributing exaggerated positive qualities to himself or others. By using narcissistic defenses, he tries to protect himself from the loss of self-esteem related to negative feelings of disappointment, powerlessness, worthlessness, and so on. Moreover, he mostly uses intellectualization to detach and distance himself from painful affects, but also rationalization and denial to refuse acknowledging some aspects of reality. Notably, Albert’s defensive profile showed the use of mature defenses (especially humor) that help him in developing better adaptation strategies; at the same time, he may use immature defenses (especially splitting of self and others and acting out). These defense mechanisms usually occur in response to painful situations and show the boy’s inability to tolerate negative affects and reflect on the events that stimulate them.

#### 3.2.4. Treatment and Outcome

After receiving an articulated diagnostic assessment (reported in the present work), Albert came to a psychodynamic treatment (one session once a week). This approach was considered the most appropriate form of treatment, taking into account his psycho(patho)logical characteristics. Overall, Albert’s psychotherapy was informed by technical guidelines following the most widely recognized psychodynamic assumptions [64], including: a focus on affect and emotional expression of emotion; exploration of attempts to avoid distressing thoughts and feelings; identification of recurring themes and patterns; discussion of past experiences; a focus on interpersonal relations; a focus on the therapeutic relationship; and an exploration of the fantasy life (for a deeper discussion of the principles of these strategies, see, e.g., [65]).

A detailed description of the course of treatment is beyond the scope of this article, but it is important to clarify that, during earlier stages of treatment, the therapist used mostly clarification to increase Albert’ awareness of their communication and to facilitate the discussion of suppressed material, but also provided supportive interventions to enhance the alliance. Then, he tried to maintain a good-enough therapeutic relationship and consistently addressed all the alliance ruptures as they arose; promoted the exploration of depressive affective states related to Albert’s loneliness, isolation, and a sense of failure strongly associated with deep disappointments and conflicts with significant others; addressed dysfunctional defense mechanisms with the aim of helping Albert develop more adaptive ways of regulating low self-esteem and feelings of inadequacy; worked to consolidate the unstable sense of Albert’s self and its poor sense of well-being, confidence, and agency related to its fragilities; identifying recurring interpersonal patterns expressed in the therapeutic relationship and linking them with patterns characterizing his current relationships, as well as early attachment experiences with his caregiver in infancy and childhood.

Albert’s psychotherapy had a good enough assessment with a disorder-specific symptom or dysfunctional behavior reduction. Overall, the psychological treatment promoted more adaptive functioning in a family and social context, and enhanced overall capacities and resources.

## 4. Discussion

The present work has sought to highlight the clinical relevance of a complex and accurate diagnosis in favoring a deeper understanding of youth depression and guiding therapeutic interventions tailored to specific characteristics of adolescent psychological functioning. Notably consistent with the PDM-2 framework, the present single case study suggests the need to explore distinct signs and symptoms of depression in the wider context of mental functioning and emerging personality patterns through an in-depth diagnostic assessment. A clinically- and empirically-informed diagnosis can help mental health professionals understand psychopathology by detecting functional processes that underlie several clinical conditions [17].

Albert self-referred recurrent depressive symptoms that seriously compromised many domains of his life. Overall, the PDC-A’s evaluation allowed to identify different difficulties and moderate levels of impairment in various mental capacities (see MA Axis in Table 2), especially in regulating self-esteem, maintaining the stability of relationships, and tolerating intense dysphoric affects. Moreover, consistent with clinical and empirical literature on depressive disorders [26,28,62], the PA Axis (see Table 2) showed that Albert presented a borderline personality organization at a high level [57] and an emerging narcissistic personality syndrome (see also Figure 1). Finally, Albert’s subjective experience of depression (SA Axis) revealed the prevalence of “introjective” characteristics [61].

On the whole, Albert’s diagnostic picture seems to confirm that his recurrent depressive falls were related to a marked vulnerability to disruptions of an effective, positive, and stable sense of self, and, consequently, expressed a painful and intolerable sense of loss of control and autonomy, accompanied by an overwhelming feeling of worthlessness and failure [66]. Albert’s concerns about self-definition and difficulties with self-esteem regulation constantly require infusions of external validation regarding their importance and value [67]. When an individual fails to recognize their intellectual brightness, cultural superiority, and physical beauty, they feel profoundly depressed, ashamed, and sometimes may fantasize about suicide to obtain some temporary, vicarious gratification by escaping from painful emotions [68].

Another relevant issue that characterizes Albert’ functioning is represented by severe problems in metabolizing anger and managing frequent outbursts. Often, depressed adolescents fear the loss of approval and acceptance from significant others, engaging them in angry tirades. These internal fights against depressive affects and attempts to avoid feeling helpless by becoming angry instead can be viewed as efforts to again conquer a sense of superiority [69,70].

Albert’s affective experience is essentially characterized by feelings of grandiosity and inadequacy, accompanied by helplessness, loneliness, shame and humiliation, and hypersensitivity to criticism [71]. The pervasive anxiety regarding threats to the self negatively influences the quality of his interpersonal relationships [72]. Indeed, as highlighted by the PDC-A (MA Axis), Albert’s capacity to engage in stable, intimate, and satisfying relationships is one of the most “damaged”. Evidence has revealed that patients presenting with a narcissistic personality show deficient emotional empathy related to their severe inability to interact with mutuality and reciprocity with others [73]. Moreover, they present impairments in mentalizing that reflect their difficulties in understanding others’ reactions and behaviors in terms of underlying internal states [74]. In their studies on attachment, Diamond and colleagues [75] have found that those patients with narcissistic and borderline personality pathologies were more likely to be categorized as either dismissing (avoidant) or “cannot classify,” whereas borderline patients were more likely to be classified as either preoccupied (anxiously attached) or unresolved for loss and abuse. However, this research supported evidence that both patients present poor mentalization.

The interpersonal difficulties of narcissistic patients tend to be recreated in a therapeutic relationship and pose great challenges to clinicians [58,76,77]. In the present study, a careful exploration of the interpersonal experience between Albert and his clinician revealed a complex and nuanced configuration of countertransference patterns that confirm clinical knowledge and empirical research findings in the field [78]. In particular, the mixed combination of special involvement in therapy and a sense of inefficacy and frustration seem to reflect core psychological dynamics of narcissistic patients. Indeed, when distressed, Albert may distort his perception of the clinician by using primitive defense mechanisms. The quick shifts between polarized affects, accompanied with the rapid fluctuations between idealization and devaluation in self- and other-representations, evoking in therapist feelings of irritation, a sense of criticism, feelings of helplessness, and withdrawal [79]. Sometimes, the therapist may feel ineffectual, colorless, invisible, and deskilled. Thus, the common defensive response to this feeling of uselessness is to become disengaged. Moreover, in these difficult situations, the clinician feels the lack of connection and trust with Albert and experiences severe problems in maintaining a good enough alliance [80]. However, the clinician also experiences greater emotional attunement and affective warmth. He recognizes that, when less upset, Albert tends to show good psychological recourses, more mature defenses (especially humor), and great commitment in treatment.

The present work shows some limitations. First, to inform and guide decisions regarding diagnosis/main problems and treatment planning of an adolescent, it would be useful to include some assessment measures focused on the functioning of the family and parents. Moreover, in line with other single case studies, the research design did not allow to find specific evidence regarding the impact of particular interventions on the patient’s functioning during the treatment process, nor regarding the effectiveness of the therapy; thus, we have to infer that Albert’s improvements were due to his treatment (and not to his history, maturation, life events, or the like). In addition, we administered several measures to the clinician and his evaluations that could reflect some biases related to social desirability or implicit defensive processes. However, we have implemented some instruments from external observers to mitigate these concerns and verify agreement among informants.

## 5. Conclusions

For decades, diagnosis has been considered a “dirty word” by several clinicians, especially psychodynamics [67]. The misuse of psychodiagnostic formulations as a sort of oversimplified labelling has contributed to cause the gradual loss of clinical value of the diagnosis. In fact, the quality of any diagnostic process is determined by its clinical application, and its ultimate goal is to facilitate better outcomes [18]. The PDM-2 aimed to integrate the descriptive and symptom-based approach to the patients’ clinical conditions, offered by the most widespread international classification systems, and a clinically sensitive, empirically-supported, and developmentally-oriented diagnostic approach. Only a careful assessment able to capture the unique qualities of each individual in idiographic terms while, at the same time, identifying the nomothetic aspects shared by groups of individuals with similar characteristics, allows for an effective treatment plan, especially in youth populations.

The present study addressed the single case of Albert, a young patient presenting with a recurrent MDD using the DSM-5 criteria [8], according to the PDM-2 clinical-diagnostic framework. Overall, the case discussion showed that, to promote comprehensive diagnoses and clinical formulations in adolescents, it is essential to assess mental functioning, styles, and emerging personality syndromes and symptom patterns, taking into account the developmental trajectories and adaptation processes that characterize this developmental period. This contribution intends to emphasize the assumption that, to understand the complexity of an adolescent’s psychological functioning and foster patient-tailored treatment, it is crucial to put the young person at the center of the diagnostic and therapeutic process and pay full attention to the developmental dynamics of the emerging mind [17].

## Figures and Tables

**Table 1:** Defense levels and their corresponding defenses.

Defense Level	Defense Mechanisms
Highly adaptive (mature)	Affiliation, altruism, anticipation, humor, self-assertion, self-observation, sublimation, suppression
Obsessional	Isolation of affect, intellectualization, undoing
Neurotic	Repression, dissociation, reaction formation, displacement
Minor image-distorting (narcissistic)	Devaluation (of self and others’ images), idealization (of self and others’ images), omnipotence
Disavowal	Denial, projection, rationalization, autistic fantasy
Major image-distorting (borderline)	Splitting (of self and others’ images), projective identification
Action	Acting out, help-rejecting complaining, passive aggression

**Table 2:** Albert’s Psychodiagnostic Chart-A (PDC-A).

Psychodiagnostic Chart-A	Scores
SECTION I: Mental Functioning (MA AXIS)	
Cognitive and affective processes	3
Capacity for regulation, attention, and learning	2
2. Capacity for affective range, communication, and understanding	3
3. Capacity for mentalization and reflective functioning	
Identity and relationships	3
4. Capacity for differentiation and integration (identity)	2
5. Capacity for relationships and intimacy	2
6. Capacity for self-esteem regulation and quality of internal experience	
Defense and coping	3
7. Capacity for impulse control and regulation	3
8. Capacity for defensive functioning	4
9. Capacity for adaptation, resiliency and strength	
Self-awareness and self-direction	3
10. Self-observing capacities (psychological mindedness)	3
11. Capacity to construct and use internal standards and ideals	2
12. Capacity for meaning and purpose	3
Overall level of personality severity (sum of 12 mental functions)	33
SECTION II: Level of Personality Organization (PA AXIS)	
Identity (ability to view self in complex, stable, and accurate ways)	5
2. Object relations (ability to maintain intimate, stable, and satisfying relationships)	4
3. Level of defenses	5
4. Reality testing (ability to appreciate conventional notions of what is realistic)	7
Overall Personality Organization	5
SECTION III: Emerging Personality Patterns/Syndromes (PA AXIS)	
Depressive	3
Anxious/Avoidant	4
Schizoid	/
Antisocial/Psychopathic	4
Narcissistic	2
Paranoid	3
Impulsive/Histrionic	3
Borderline	3
Dependent/Victimized	/
Obsessional	4
SECTION IV: Symptom Patterns (SA AXIS)	
Symptom/Concern: Recurrent Major Depressive Disorder Level:	3
SECTION V: Cultural, Contextual and Other Relevant Considerations	

Albert is the eldest son of a middle class family. His mother is a teacher in a secondary school and his father is a rather successful artist. The boy refers to having always breathed in a climate of exclusivity linked to his father’s aspiration, and describes how both his parents seem to have had a special investment with him.

## Data Availability

Not applicable.

## Supplementary Materials

The following are available online at https://www.mdpi.com/article/10.3390/ijerph181910127/s1, Table S1: Albert’s emerging personality profile derived from the SWAP-II-A, Table S2: Albert’s defensive profile derived from the DMRS-Q.
