# Supplementary material for: From a Symptom-Based to a Person-Centered Approach in Treating Depressive Disorders in Adolescence: A Clinical Case Formulation Using the Psychodynamic Diagnostic Manual (PDM-2)’s Framework
Source: Int J Environ Res Public Health. 2021 Sep 27;18(19):10127. doi: 10.3390/ijerph181910127 (PMC8508312; doi:10.3390/ijerph181910127)
Supplement: Supplementary file 1 [file ijerph-18-10127-s001.zip › ijerph-1356757-supplementary.pdf]

International Journal of
Environmental Research and Public Health

MDPI

Table S1. Supplementary material related to Figure 1: Albert's emerging personality profile derived from the SWAP-II-A

SWAP-II-A[a] PD[b] scales	Scores
Paranoid	51.16
Schizoid	50.22
Schizotypal	43.30
Antisocial	47.50
Borderline	57.12
Histrionic	52.06
Narcissistic	63.74
Avoidant	53.20
Dependent	35.12
Obsessive-Compulsive	55.85
High-Functioning	53.40

[a] SWAP-II-A = Shedler-Westen Assessment Procedure for Adolescents, Version II-A; [b] PD scales = Personality Disorders scales.

Table S2. Supplementary material related to Figure 2: Albert's defensive profile derived from the DMRS-Q

DMRS-Q[a]	Scores
Overall defensive functioning	4.15
Defense levels	
High–adaptive	13.10
Obsessional	16.67
Neurotic	14.29
Minor–image distorting	19.05
Disavowal	14.29
Major–image distorting	10.71
Action	11.90
Defense mechanisms	
Suppression	0.00
Sublimation	0.00
Self–observation	3.57
Self–assertion	3.57
Humor	5.95
Anticipation	0.00
Altruism	0.00
Affiliation	0.00
Isolation of affects	4.76
Intellectualization	7.14
Undoing	4.76
Repression	3.57
Dissociation	3.57
Reaction Formation	4.76
Displacement	2.38
Devaluation of self–image	3.57
Devaluation of others' image	3.57
Idealization of self–image	3.57
Idealization of others' image	2.38
Omnipotence	5.95
Denial	2.38
Rationalization	7.14
Projection	2.38
Autistic fantasy	2.38
Splitting of self–image	4.76
Splitting of others' image	3.57
Projective identification	2.38
Passive aggression	3.57
Help–rejecting complaining	2.38
Acting out	5.95

[a] DMRS-Q = Defense Mechanism Rating Scale-Q sort
